# Postinsertion iliac vein stent position and relationship to contralateral deep vein thrombosis after unilateral iliac vein stenting

**DOI:** 10.1016/j.jvsv.2026.102577

**Published:** 2026-07-10

**Authors:** Anusha Jacob, Claudia Theis, Alexis Betancourt, Daniel Calderon, William A. Marston

**Affiliations:** Division of Vascular Surgery, Department of Surgery, The University of North Carolina School of Medicine, Chapel Hill, NC

**Keywords:** Iliofemoral deep venous thrombosis, Post thrombotic syndrome, Venous stenting

## Abstract

**Objective:**

Nitinol stents designed for the treatment of iliac venous obstructive lesions were expected to improve shortcomings associated with previously available stents, including accuracy of deployment at the iliac confluence and insufficient stent expansion at the site of compressive obstruction. Comparative studies to measure these characteristics have not been reported. This study was performed to measure the position of venous stents after insertion and the effect of coverage of the contralateral iliac orifice, comparing nitinol venous (NV) stents to Elgiloy stents (Boston Scientific Corporation; WALLSTENT Endoprosthesis).

**Methods:**

Patients with a history of iliac venous stent insertion where the proximal end of the stent was placed at the iliac confluence were identified retrospectively. Patients studied with a poststent computed tomography/magnetic resonance scan that allowed examination of the position of the previously placed stent were selected for study inclusion. Imaging was reviewed using three-dimensional reconstructions to determine the accuracy of stent deployment at the iliac confluence and the position of the stent relative to the contralateral iliac orifice. The incidence of residual iliac obstruction after stent insertion, and the incidence of poststent ipsilateral or contralateral thrombosis was determined.

**Results:**

One hundred sixty-three patients with iliac vein stent were identified with poststent axial imaging with a median age of 61 years. In total, 66.3% patients were female, and 73.6% were Caucasian. Stent insertion was performed on the left side in 87.1% of cases. Stenting was performed with Elgiloy stents in 36.8% of cases and NV stents in 63.2% with median follow-up of 3.3 ± 3.4 years. In 64 cases (39.3%), stents completely extended across the entire (100%) contralateral iliac orifice, with no significant difference in incidence between Elgiloy stents (31.7%) and NV stents (43.7%). Persistent stent compression >25% at the confluence was seen in 9.8% of Elgiloy stents and 6.8% of NV stents. Contralateral iliac vein thrombosis after stenting occurred in 8.0% of patients at 5 years and was statistically more frequent in those where the iliac stent was placed completely across the contralateral iliac orifice (*P* = .046). On Cox regression adjusted for limb laterality, each 10 percentage-point increase in orifice obstruction was associated with a 61% increase in the hazard of contralateral deep vein thrombosis (hazard ratio, 1.61; 95% confidence interval, 1.20-2.15; *P* = .001). Stent type was not associated with the incidence of contralateral iliac deep vein thrombosis.

**Conclusions:**

Complete coverage of the contralateral iliac orifice was found in almost 40% of patients after unilateral iliac vein stenting. The use of NV stents did not result in a decreased incidence of contralateral coverage. Contralateral iliac vein thrombosis was more frequent after complete coverage of the contralateral iliac orifice suggesting that this should be avoided at the time of stent insertion. Long-term follow up is required to fully determine the incidence of this complication and its relationship to stent type or stent positioning.

**Article Highlights**

• **Type of Research:** Single center retrospective study
• **Key Findings:** In this cohort of 163 patients, the association of iliac vein stent position and poststent thrombosis was examined. Over 40% of stents were placed completely across the contralateral iliac vein orifice and this was associated with an increased risk of contralateral deep vein thrombosis.
• **Take Home Message:** Complete coverage of the contralateral iliac vein orifice should be avoided when treating unilateral iliac vein disease.

Treatment of chronic post-thrombotic (PT) obstruction or compression of the iliocaval venous system using intravenous stents has been validated as a useful technique to reduce symptoms related to poor outflow of venous blood.^1^^,^^2^ As reported by Neglen et al,^3^ venous stenting was initially performed using stents designed for use in the biliary or arterial systems. Although favorable outcomes with symptom improvement were reported using these stents, some shortcomings in stent characteristics and performance were identified.

The most common stent used before the development of venous nitinol stents was the Wallstent (Boston Scientific Inc). This stent, a self-expanding design constructed of Elgiloy, was useful due to its flexibility and compression resistance, particularly in the body of the stent. However, the design was noted to have significantly less compression resistance at the ends, if the ends were not fixed in position.^4^^,^^5^ This led to a risk of persistent venous stenosis at the site of arterial compression if the stent was not inserted several centimeters into the inferior vena cava (IVC). Placement of the stent into the IVC for unilateral cases resulted in jailing the contralateral iliac orifice at the iliac confluence. Reports of a pseudointima covering the stent interstices across the contralateral iliac orifice suggested this as the mechanism leading to contralateral deep vein thrombosis (DVT).^6^ Although contralateral DVT was initially reported as a rare occurrence, later reports with longer patient follow-up identified contralateral DVT to occur in as many as 10% of cases after unilateral treatment with the Wallstent.^5^^,^^7^^,^^8^

To reduce the risk of contralateral DVT while eliminating compression of the proximal left common iliac vein (CIV), stent placement is generally recommended with the proximal edge of the stent landing approximately 1 cm above the area of compression, but not across the entire contralateral iliac orifice.^9^ Attempting to place the Wallstent in a precise location can be challenging due the deployment mechanism and significant shortening of the stent during deployment. Other technical modifications have been recommended, including the hybrid Wallstent/Z-stent technique, designed to avoid blockage of the contralateral iliac vein while maintaining robust compression resistance. The results using this configuration were reported with favorable outcomes; however, this required the use of off-label stents not specifically designed to be used together.^10^

The development of nitinol venous (NV) stents aimed to address these and other shortcomings by offering a stent with high resistance to compression at the ends of the stent while maintaining acceptable flexibility to accommodate the tortuosity of the iliac veins in the pelvis.^11^^,^^12^ Nitinol stents shorten significantly less than Elgiloy stents at deployment. This characteristic, combined with the use of triaxial deployment systems was expected to improve accuracy of deployment.^12^^,^^13^ In theory, these factors would allow the treatment of proximal CIV compression without requiring insertion into the IVC. It was believed that stent placement only partially covering the contralateral iliac vein orifice combined with larger stent interstices should reduce the incidence of contralateral venous thrombosis. However, these potential benefits have not been proven in clinical use, and some data suggests that different stent designs may not yield significantly different outcomes in real-world use.^14^

The aim of this study is to evaluate the position of stent placement in patients after unilateral venous stenting with Elgiloy or nitinol stents as demonstrated on poststent abdominal/pelvic cross-sectional imaging. We also studied the incidence of persistent stent compression and contralateral DVT and their association with stent positioning.

## Methods

A retrospective database was used to identify patients with unilateral iliac vein stent insertion performed between 2005 and 2024, in whom the proximal end of the stent was placed at the iliac confluence. Candidates were selected for study inclusion if they also had poststent computed tomography (CT) or magnetic resonance imaging (MRI) of the abdomen/pelvis that allowed examination of the position of the previously placed stent. Patients with IVC stents, kissing iliac vein stents or any other bilateral iliac vein interventions were excluded from the study (Table I). Patient information was reviewed to define demographic factors (such as sex, race, and ethnicity), body mass index, and history of acute DVT, PT syndrome, or nonthrombotic (NT) iliac compression. Two groups of patients were selected for comparison; those who had Elgiloy stents placed (EG group) and those who had NV group stents placed.

**Table I:** Study inclusion/exclusion criteria

Patients were enrolled through inquiry of prospectively maintained database of all patients being followed for previously placed iliocaval venous stents
Inclusion criteria:
• All patients undergoing unilateral iliac vein stent insertion by any physician within study health care system^a^
• Poststent CT or MRI for any reason of sufficient quality to identify stent positioning at iliac venous confluence
Exclusion criteria:
• Inferior vena cava stent
• Kissing iliac vein stents or other bilateral iliac vein interventions
• Iliac stent inserted >0.5 cm distal to iliac vein confluence

*CT*, Computed tomography; *MRI*, magnetic resonance imaging.

a Some patients had initial stent placed at facility outside of authors' institution and then referred for stent follow-up.

### Measurement of iliac stent positioning

**Fig 1:**
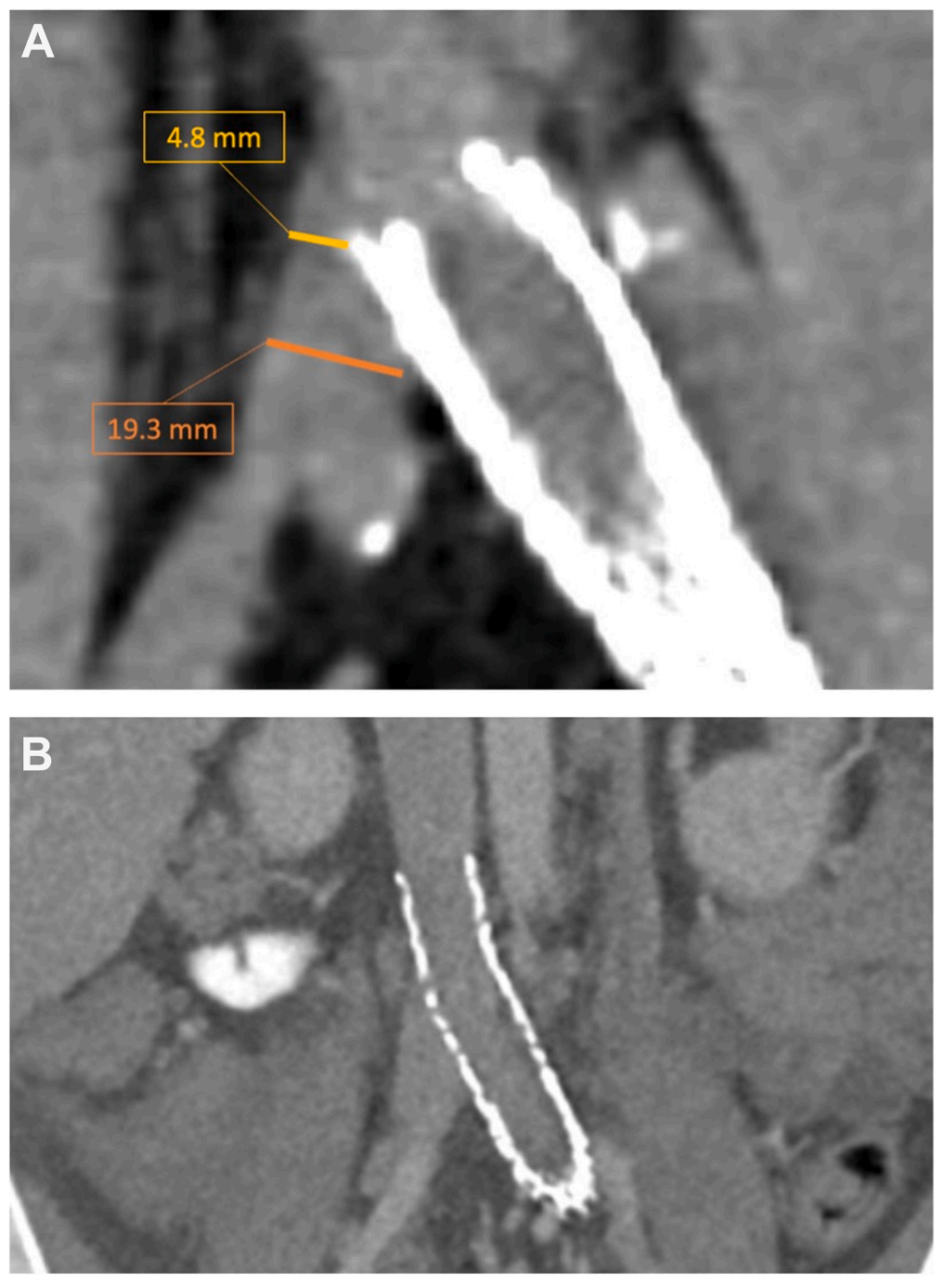
Determination of degree of overlap of contralateral iliac vein orifice on computed tomography (CT) scan. **A,** Total right iliac diameter just below confluence (19.3 mm) and unobstructed length of iliac vein at confluence (4.8 mm) yields calculation of 75.1% overlap. **B,** 100% overlap of contralateral iliac vein orifice with stent placed to opposite wall of inferior vena cava (IVC).

Imaging completed poststent insertion was reviewed using three-dimensional reconstructions to determine the accuracy of stent deployment at the iliac confluence. CT and MRI images were reviewed using the same protocol. If images were not sufficiently clear to accurately measure the required datapoints, the patient was excluded from the study. Axial images were compared with linked coronal images side by side to determine the location of the iliac vein stent at the venous confluence. Axial images were scanned to identify the proximal edge of the stent. The linked coronal images were then scanned around this location to identify the image that best displayed the proximal edge of the stent and its position related to the contralateral iliac orifice and the walls of the IVC as the right CIV and left CIV merged. The distance of overlap for the iliac vein stent into the contralateral iliac orifice, and the diameter of the contralateral iliac vein were measured in millimeters in the coronal view of imaging as represented in Fig 1, *A*. Fig 1, *B* illustrates a stent with complete coverage of the contralateral iliac orifice. These measurements were then used to determine the percent of overlap of the iliac stent into the contralateral iliac vein after stent insertion. The overlap percentage was calculated by dividing the stent overlap distance by the diameter of the contralateral iliac orifice and multiplied by 100.

The reproducibility of measurement of the percentage of contralateral iliac coverage using the described methodology was studied in a subset of 50 CT scans. Two independent observers performed image analysis and calculations. The overlap variability was assessed by determining the interobserver correlation coefficient (A, 1) using a two-way random-effects absolute agreement model, with Bland-Altman analysis performed to assess systematic bias and limits of agreement.

The degree of persistent stenosis of the stented iliac vein was determined by measuring the diameter of the stented CIV at the point of maximal compression compared with the diameter of the stented CIV distal to the point of compression. Patient imaging and records were analyzed for the prevalence of persistent proximal iliac vein stent compression, contralateral iliac vein thrombosis and/or ipsilateral iliac vein stent thrombosis or stenosis >50% requiring repeat intervention.

### Statistical analysis

Continuous variables are presented as median (range) or means ± standard deviation, as appropriate. Demographics and variables between groups were compared with *χ*^2^ testing or the Mann-Whitney *U* test. The cumulative probability of contralateral thrombosis over time is determined using Kaplan-Meier product-moment estimates. Log-rank testing is used to compare the incidence of events occurring over time in separate groups. Cox proportional hazards regression was used to evaluate predictors of time to contralateral iliac vein thrombosis, with percentage contralateral orifice obstruction entered as a continuous variable per 10 percentage-point increment. Covariates were selected a priori based on clinical plausibility; final model selection was guided by the Akaike Information Criterion. Given the limited event count (n = 11), a maximum of two covariates were included in the multivariable model. Firth penalized Cox regression was performed as a sensitivity analysis. Logistic regression was used to identify predictors of complete contralateral orifice coverage. All analyses were performed using R version 4.5.2 (R Foundation for Statistical Computing).

The institutional review board of the University of North Carolina approved this study and determined informed patient consent was not needed because of the retrospective nature of the study.

## Results

One hundred sixty-three patients were identified with a history of unilateral iliac vein stenting of the CIV with or without more distal stenting who also had CT (n = 161) or MRI (n = 2) imaging performed of sufficient quality to determine the positioning of the proximal iliac stent. The indications for CT or MRI are listed in Table II, with approximately half being performed for venous indications. The median age was 61 years (range, 11-96), 66.3% were female and 73.6% were white. Stenting was performed for acute DVT in 32 (19.6%) cases, NT venous compression in 58 (35.6%), and PT venous obstruction in 73 (44.8%) cases. The anatomic extent of venous obstruction requiring stenting are listed using the classification system described by Jalaie et al^15^ (Table III). Stent insertion was performed on the left side in 87.1% of cases. Stenting was performed with Elgiloy stents in 36.8% of cases and NV stents in 63.2% with median follow-up of 3.3 ± 3.4 years. Median follow-up was significantly longer for Elgiloy stents (62.4 months) than for NV stents (24.4 months, *P* < .01). The use of intravascular ultrasound (IVUS) guidance was documented at the index stenting procedure in 92% of cases.

**Table II:** Indication for poststent computed tomography (CT)/magnetic resonance imaging (MRI)

Indications	Patients (%)
Venous	49.7
Abdominal pain	26.4
Cancer staging	4.3
Renal	4.9
Miscellaneous	14.7

Categorical data are shown as number (%).

**Table III:** Etiology and anatomic type of iliac vein obstruction

Etiology of venous obstruction	Total = 163
Nonthrombotic	58 (35.5)
Post-thrombotic	73 (44.8)
Acute	32 (19.6)

Classification of iliofemoral chronic venous obstruction^a^	Patients (%)
Type 1	42.3
Type 2	20.2
Type 3	22.1
Type 4/5	15.3

Categorical data are shown as number (%).

a Classification per Jalaie et al.^15^

The reliability of the method used to calculate the degree of coverage of the contralateral iliac orifice was excellent, with an interobserver intraclass correlation coefficient of interobserver correlation coefficient (A, 1) = 0.974 [95% confidence interval (CI), 0.950-0.986]. Bland-Altman analysis demonstrated a mean bias of 2.9% with limits of agreement of −11.7% to +17.5%. On poststent CT/MRI, the proximal portion of the stent extended across the entire (100%) contralateral iliac orifice in 64 cases (39.3%). The incidence of complete contralateral iliac orifice coverage did not differ significantly between Elgiloy stents (31.7%) and NV stents (43.7%) (Table IV). Persistent narrowing of the left CIV as it passed under the right common iliac artery was assessed after stenting in the 136 cases where stents were placed on the left side with no hybrid or overlapping stents in the proximal segment. Persistent stent compression >25% at the confluence was seen in 9.8% of Elgiloy stents and 6.0% of all NV stents, which was not significantly different. More severe narrowing >50% was found in only three cases, with no relationship to the type of iliac stent. In this patient cohort, stent restenosis or occlusion requiring reintervention occurred in 19 patients (11.7%) at a mean follow-up of 3.3 years. Stent stenosis or occlusion occurred in 4 of 13 (30.7%) patients with persistent poststent narrowing compared with 15 of 123 (12.2%) without stent narrowing. The difference was not statistically significant (*P* = .066).

**Table IV:** Incidence of Elgiloy vs Nitinol stent covering entire contralateral iliac orifice

Variable	Elgiloy (N = 60)	Nitinol venous (N = 103)	*P* value
Proximal stent overlapping entire (100%) contralateral iliac orifice	31.7	43.7	.14 (NS)

*NS*, Nonsignificant.

Categorical data are shown as number (%).

**Fig 2:**
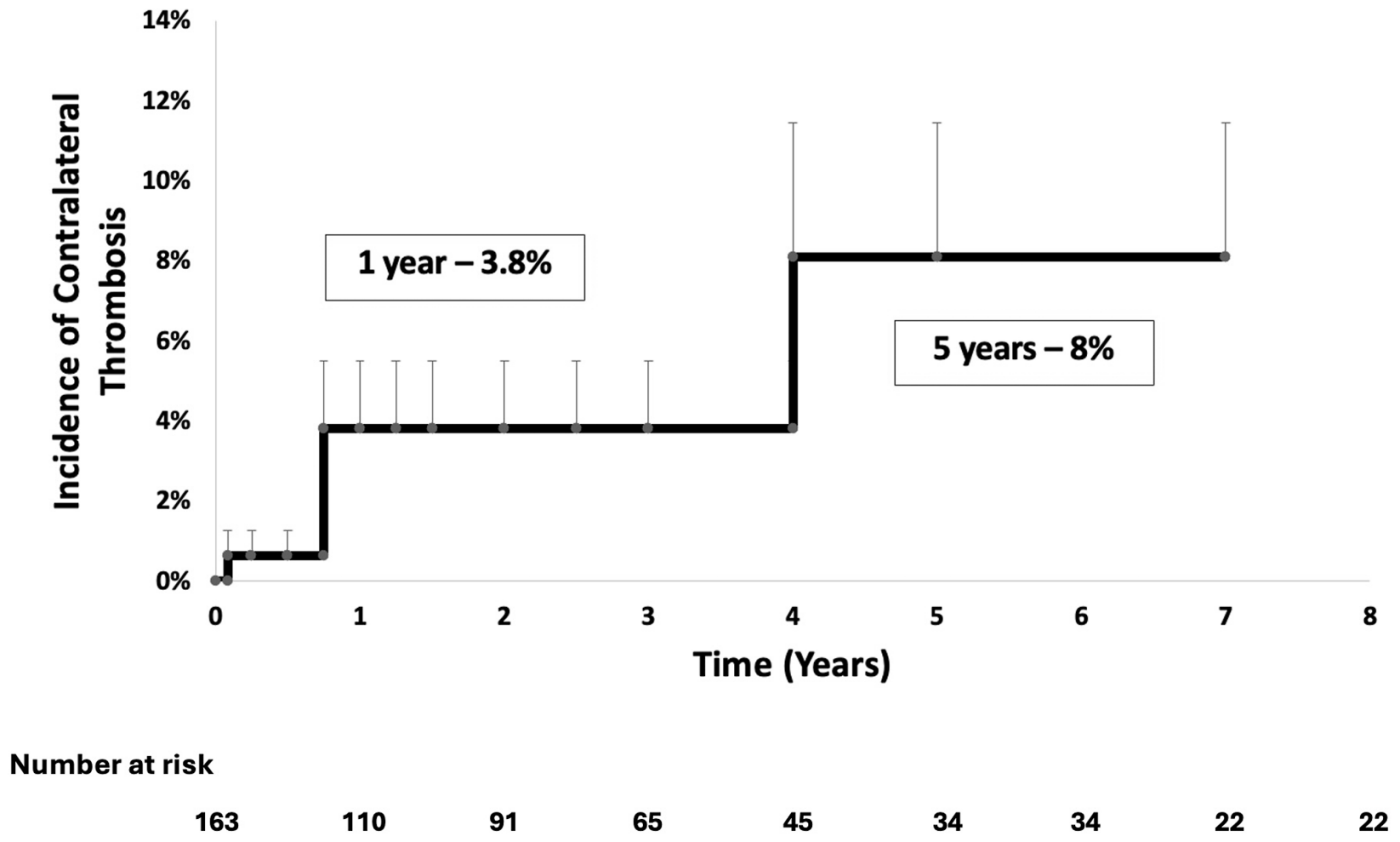
Incidence of contralateral iliac vein thrombosis poststent deployment.

**Fig 3:**
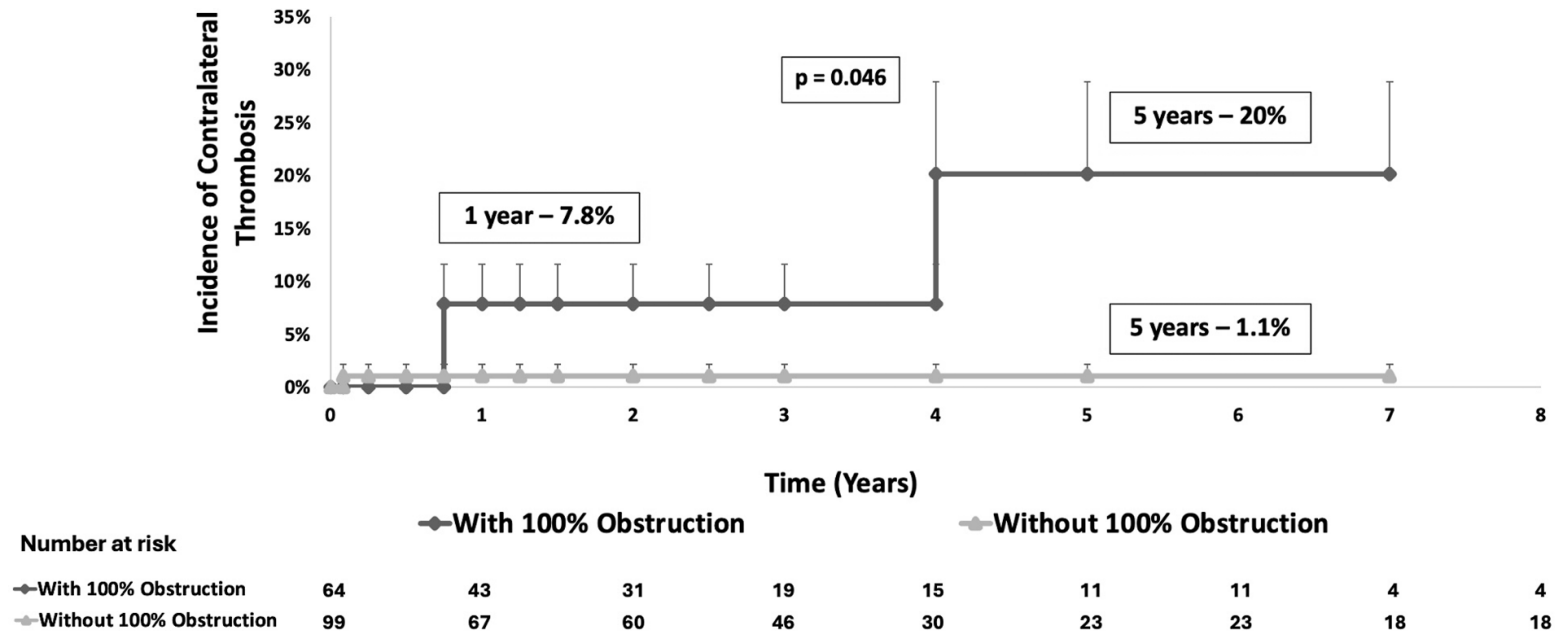
Incidence of contralateral iliac vein thrombosis in patients with and without 100% obstruction of contralateral iliac orifice.

Contralateral DVT after stenting occurred in 11 (6.8%) patients at a median time of 30.1 months after initial stent placement. Eight of these DVT involved the contralateral iliac vein up to the iliac confluence. By Kaplan-Meier life table analysis, the incidence of contralateral DVT was 3.8% at 3 years and 8.0% at 5 years (Fig 2). Contralateral thrombosis did not appear to depend on the type of venous disease, occurring after stenting for cases of NT, PT disease, and after acute thrombosis. Contralateral iliac DVT occurred after stenting with two Elgiloy and six NV stents, with no statistical association between stent type and DVT occurrence. Seven of the eight cases of contralateral iliac DVT occurred in patients where the initial iliac stent was placed covering 100% of the iliac orifice. In this group, the incidence by life table analysis was 7.8% at 3 years and 20.0% at 5 years, which was significantly higher than the incidence in those that did not have complete coverage of the contralateral orifice (*P* = .046; Fig 3). Notably, 50% of cases of contralateral thrombosis occurred in patients who had a known hypercoagulable state despite the use of long-term anticoagulation.

On Cox proportional hazards regression adjusted for limb laterality, each 10 percentage-point increase in contralateral orifice obstruction was independently associated with a 61% increase in the hazard of contralateral iliac vein thrombosis (hazard ratio, 1.61; 95% CI, 1.20-2.15; *P* = .001; Table V), confirmed on Firth-penalized Cox regression as a sensitivity analysis (hazard ratio, 1.54; 95% CI, 1.22-2.13; *P* < .001). Stent type was not significantly associated with contralateral DVT [NV vs Wallstent: odds ratio (OR), 1.47; 95% CI, 0.37-7.18; *P* = .596]. On logistic regression, the variables associated with complete contralateral iliac orifice coverage were left-sided stenting (OR, 7.29; 95% CI, 1.90-48.5; *P* = .012) and younger age (OR, 0.98/year; 95% CI, 0.96-1.00; *P* = .031; Table VI).

**Table V:** Cox proportional hazards regression: Predictors of contralateral iliac vein thrombosis

Model	Variables	HR	95% CI	*P* value
All contralateral DVT (n = 11 events)				
Adjusted^a^ (final)	% Obstruction (per 10%)	1.61	1.20-2.15	.001
Iliac DVT only—sensitivity (n = 8 events)				
Univariate	% Obstruction (per 10%)	1.82	1.12-2.97	.016
Adjusted^a^	% Obstruction (per 10%)	1.87	1.15-3.03	.011
Adjusted^a^	Left limb (vs right)	0.19	0.02-1.92	.161

*CI*, Confidence interval; *DVT*, deep vein thrombosis; *HR*, hazard ratio.

a Adjusted model includes % contralateral orifice obstruction and limb laterality. Firth-penalized Cox sensitivity: (HR, 1.54; 95% CI, 1.22-2.13; *P* < .001).

**Table VI:** Logistic regression: Predictors of complete contralateral orifice coverage (100% obstruction) and contralateral deep vein thrombosis (DVT)

Variables	OR	95% CI	*P* value
Age, per year	0.98	0.96-1.00	.031
Left limb (vs right)	7.29	1.90-48.5	.012
Female sex	0.91	0.43-1.90	.794
BMI, per unit	0.97	0.93-1.02	.256
IVUS guidance	1.48	0.41-5.76	.552

*BMI*, Body mass index; *CI*, confidence interval; *IVUS*, intravascular ultrasound; *OR*, odds ratio.

N = 163, 64 patients (39.3%) had complete orifice coverage.

## Discussion

Treatment of iliocaval venous obstruction in patients with pelvic and lower-extremity symptoms continues to evolve as new devices are developed and integrated into clinical practice. Accurate deployment of venous stents is important to provide complete coverage of diseased or compressed venous structures while limiting the risk of stent migration or compromise of normal surrounding venous branches. In their initial descriptions of iliac vein stenting using primarily the Wallstent, a stent constructed of Elgiloy, Neglen et al^3^ recommended that the stent should be placed well into the IVC to successfully open the area of obstruction and prevent early restenosis. Placement of the Wallstent up into the IVC was initially reported to rarely result in contralateral iliac flow problems due to coverage of the orifice.

After longer duration of patient follow-up, the same group reported a higher incidence of contralateral DVT after Wallstent insertion, introducing a modification using Gianturco Z-stents (Cook Medical) at the upper end of the Wallstent at the confluence to mitigate the issues noted above.^16^ Murphy et al^5^ described contralateral iliac thrombosis occurring in approximately 10% of cases after unilateral iliac stenting with the Wallstent compared with 1% when using the Gianturco Z-stent modification. It is notable that the average time after stenting of contralateral DVT occurrence in their series was 47.5 months.

Rathore et al^6^ reported a case of surgical explantation of a Wallstent for chronic pain that was found to have formed a pseudointima around the portion of the stent placed across the contralateral common iliac orifice. Explantation was performed 2.5 years after stent implantation. The patient did not have right lower-extremity symptoms at the time of surgery but some of the pores of the portion of the stent placed across the right iliac orifice were found to be covered with pseudointima and thrombus had formed on the stent surface limiting flow from the right lower extremity. This required excision at the time of stent explantation to re-establish normal right iliac vein outflow.

Other groups reporting on the incidence of contralateral DVT after venous stenting included Avgerinos et al^7^ who reported an incidence of 8.0% occurring at an average of 27.4 months after the initial stent placement, and Le at al^17^ who reported an incidence of 9% occurring at a median time of 40 months. Kim et al^18^ reported an incidence of contralateral DVT occurring in 5.4% of patients after iliac stenting, occurring at a median of 26 months after the index procedure. In this study, significant factors associated with the occurrence of contralateral DVT were extension of the stent into the IVC and in-stent thrombosis.

The studies reported above involved follow-up of patients treated primarily with Elgiloy stents. Nguyen et al^19^ recently reported follow-up on 409 patients who underwent iliac vein stenting with Venovo nitinol stents (Bard Peripheral Vascular, Inc.) with a low contralateral DVT rate of 0.49%. However, the median follow-up in this study was limited at 15.35 months leading the authors to conclude that contralateral DVT after nitinol stents is very low in the short term, but long-term follow-up is needed to determine the incidence and its impact on patients undergoing venous stenting, many of whom are very young.

NV stents developed with an open cell design have larger pores than Elgiloy stents. Also, improved deployment systems and less stent shortening during deployment was expected to improve the accuracy of stent deployment to limit the incidence of contralateral thrombosis.^9^^,^^20^ In our dataset, we enrolled patients with Elgiloy stents and a variety of nitinol stents with a median follow-up of 3.3 years. We identified contralateral iliac vein thrombosis in 3.8% at 3 years and 8.0% at 5 years by life table analysis. The incidence was similar in Elgiloy stents compared with NV stents by life table analysis; however, the event rate is likely inadequate to critically test the effect of stent type on contralateral DVT. Regardless, NV stents did not appear to protect against the occurrence of contralateral DVT, as there were more events in the NV group, despite a shorter length of follow-up. Most cases of contralateral iliac thrombosis occurred after the stent was placed completely across the contralateral iliac orifice, with the incidence significantly greater than in those where the stent did not completely cover the contralateral iliac. This suggests that in cases where iliocaval extension is not required by obstruction of the IVC, interventionalists should attempt to avoid complete coverage of the contralateral iliac orifice. When more proximal extension is required, bilateral iliocaval stenting should be considered.

Our findings suggest that the insertion of nitinol iliac vein stents did not achieve increased accuracy compared to Elgiloy stents, as measured by the number of stents achieving a position of partial coverage of the contralateral iliac orifice. The number of stents placed completely across the contralateral orifice was higher but not significantly different in patients treated with nitinol stents. Based on the study methodology, we were not able to determine the intended proximal deployment location for each stent, or the difference in stent location immediately after deployment compared with the location months or years later at the time of CT/MRI. We excluded patients with prestent evidence of obstruction of the distal IVC or contralateral iliac vein disease from the study, but it is possible that some cases had evidence of obstruction of the distal IVC on IVUS guidance requiring higher stent placement for complete re-expansion of the flow channel.

IVUS guidance has previously been reported as a useful tool to guide stent deployment for coverage of the diseased segment of vein while minimizing needless coverage of normal vein segments.^21^ In this review, almost all cases were performed with the use of IVUS for guidance. It is possible that the accuracy of stent deployment is related to the interventionalist's method of identifying the correct location as much as the deployment mechanism or characteristics of the stent at the time of deployment.^9^

It is our observation that the most important information to guide proximal iliac stent placement at the iliac confluence is the location of the IVC bifurcation and the location of the opposite wall of the IVC just at the confluence. In most cases, the location of the IVC bifurcation can be identified with IVUS guidance, but the opposite wall of the IVC is not always within the IVUS guidance field of view. The use of additional fluoroscopy images with a catheter placed at the bifurcation and close attention to bony landmarks can assist in accurate stent placement as described by Bajwa et al.^9^

Persistent obstruction with incomplete expansion of the stent at the site of proximal compression occurred in a minority of cases, but did not statistically affect stent patency. This finding requires additional study in a larger sample followed over time such that the event rate is sufficient to adequately power statistical analysis.

It is notable that the majority of ipsilateral or contralateral thrombotic events occurring after venous stenting occurred more than 2.5 years after the stenting procedure. Long-term follow-up of patients is required to monitor for events. Additional study, particularly with newer venous nitinol stents is required to accurately characterize the risk of these events.

Significant limitations associated with this study include the retrospective design, the selection of patients based on those who underwent CT scan to allow assessment of the position and characteristics of previously placed stents, and the length of follow-up. The incidence of outcome events might be higher in patients who underwent CT scan than in those that did not, resulting in overestimation of the event rate. The low number of thrombotic events limited the complexity and stability of multivariable modeling. Although there were a significant group of patients followed for longer than 3 years, there were fewer in the NV group than in the Elgiloy group limiting the certainty of comparative evaluations between stent types.

## Conclusions

Venous stents inserted to treat obstruction of the left CIV are frequently placed across the contralateral iliac orifice, which may obstruct outflow from the right iliac vein. NV stents were no more accurate than Elgiloy stents, with complete coverage of the contralateral iliac orifice occurring in 43.7% of cases. Contralateral iliac vein thrombosis was identified in 8% of patients occurring at a median of 30 months after stenting, was not less frequent after insertion of NV stents, and was significantly more frequent in cases with complete coverage of the contralateral iliac orifice. Coverage of the entire contralateral iliac vein orifice should be avoided when not necessary due to this association with contralateral DVT. Long-time follow-up is required to fully determine the incidence of poststent thrombosis and its relationship to stent type or stent positioning. Improved iliac stent design and deployment methods are needed to facilitate accurate implantation at the iliac confluence.

## Author Contributions

Conception and design: AJ, AB, DC, WM

Analysis and interpretation: AJ, CT, AB, DC, WM

Data collection: AJ, AB, DC, WM

Writing the article: AJ, CT, AB, DC, WM

Critical revision of the article: AJ, CT, AB, DC, WM

Final approval of the article: AJ, CT, AB, DC, WM

Statistical analysis: AJ, CT, WM

Obtained funding: WM

Overall responsibility: WM

## Disclosures

W.M. has received consulting fees for scientific consulting from Intervene, Inc., and W. L. Gore & Associates, Inc. A.J., C.T., A.B., and D.C. declare no competing interests.
